# Patterns of Hemodialysis Catheter Dysfunction Defined According to National Kidney Foundation Guidelines As Blood Flow <300 mL/min

**DOI:** 10.4061/2011/891259

**Published:** 2011-12-08

**Authors:** Robert I. Griffiths, Britt B. Newsome, Geoffrey A. Block, Robert J. Herbert, Mark D. Danese

**Affiliations:** ^1^Department of Epidemiology, Outcomes Insights, Inc., Westlake Village, CA 91362, USA; ^2^Division of General Internal Medicine, Johns Hopkins University School of Medicine, Baltimore, MD 21205-2109, USA; ^3^Department of Clinical Research, Denver Nephrologists, Denver, CO 80230, USA; ^4^Department of Health Policy and Management, Johns Hopkins Bloomberg School of Public Health, Baltimore, MD 21205-2103, USA

## Abstract

Blood flow rate (BFR) <300 mL/min commonly is used to define hemodialysis catheter dysfunction and the need for interventions to prevent complications. The objective of this study was to describe patterns of unplanned BFR <300 mL/min during catheter hemodialysis using data from DaVita dialysis facilities and the United States Renal Data System. Patients were included if they received at least eight weeks of hemodialysis exclusively through a catheter between 08/04 and 12/06, and catheter hemodialysis was the first treatment modality following diagnosis of end-stage renal disease (first access), or it immediately followed at least one 30-day period of dialysis exclusively through a fistula or graft (replacement access). Actual BFR <300 mL/min despite a planned BFR ≥300 mL/min defined catheter dysfunction during each dialysis session. There were 3,364 patients, 268,363 catheter dialysis sessions, and 19,118 (7.1%) sessions with catheter dysfunction. Almost two-thirds of patients had ≥1 catheter dysfunction session, and 30% had ≥1 catheter dysfunction session per month. Patients with catheter as a replacement access had a higher rate of catheter dysfunction than those with a catheter as first access (hazard ratio: 1.13; *P* = 0.04). Catheter dysfunction affects almost one-third of catheter dialysis patients each month and two-thirds overall.

## 1. Introduction

Hemodialysis catheter dysfunction often is defined as blood flow rate (BFR) <300 mL/min [1], including in the National Kidney Foundation's Kidney Disease Outcome Quality Initiative (NKF-KDOQI) clinical practice guidelines [2]. Other definitions of catheter dysfunction reported in the literature include frequent arterial and venous pressure alarms, poor conductance, and poor dialysis efficiency based on urea reduction ratio or Kt/V calculations [3–8]. Among these definitions, the one recommended by NKF-KDOQI may be of particular significance to providers and payers. This is especially true in the United States, where NKF-KDOQI guidelines play a prominent role in shaping clinical practice, including through the Centers for Medicare and Medicaid Services' (CMS) End-Stage Renal Disease (ESRD) Clinical Performance Measures Project [9]. Since the recommendation to define catheter dysfunction as BFR <300 mL/min was opinion based, concerns have been raised that it has been interpreted to mean BFR must be kept above 300 mL/min to maintain adequate dialysis. However, one recent study showed that mean blood flows <300 mL/min were not commonly associated with dialysis inadequacy [1], prompting the authors to conclude that this definition of catheter dysfunction could result in a significant number of unnecessary interventions.

Presently, there is very little information on the epidemiology of hemodialysis catheter dysfunction defined as BFR <300 mL/min. One exception is a study by Moist and colleagues on the association between BFR and dialysis adequacy, which found that mean blood flow <300 mL/min occurred in 10% of patients [1]. However, this was a cross-sectional study of only 259 patients conducted at two university-based tertiary hemodialysis care programs. Data on BFR are not present in the United States Renal Data System (USRDS), and they are not collected as part of the CMS ESRD Clinical Performance Measures Project. Without first understanding the epidemiology of catheter dysfunction defined according to NKF-KDOQI guidelines, it is difficult to assess the potential impact of this guideline on clinical outcomes and on interventions, both necessary and unnecessary.

The objective of this study was to describe patterns of hemodialysis catheter dysfunction, defined as unplanned BFR <300 mL/min, in a large cohort of ESRD patients.

## 2. Methods

### 2.1. Study Design and Setting

An observational cohort study was performed using clinical and administrative data from DaVita Inc., merged with administrative and Medicare claims data from the USRDS. DaVita owns and operates more than 1,400 outpatient dialysis facilities in the United States and has acute units in more than 700 hospitals. Facilities are located in 43 states and the District of Columbia. Nationwide, DaVita serves approximately 110,000 patients. The DaVita clinical data warehouse is a repository for detailed demographic, treatment, medication, and laboratory information. Information is available for each patient's individual dialysis sessions, allowing the investigator to reconstruct detailed longitudinal treatment histories.

The USRDS is a national data system that collects, analyzes, and distributes information about ESRD in the United States [10]. It contains demographic, diagnosis, and treatment history information for all Medicare beneficiaries with ESRD, a point-prevalent cohort of approximately 570,000 in the second quarter of 2009 [11]. Also, it contains 100% of Medicare inpatient and outpatient bills for these patients. Presently, Medicare Part D oral medication claims are not included.

The data set used in this project was composed of a point-prevalent dialysis patient population in August 2004, with a maximum follow-up period through December 31, 2006.

### 2.2. Participants

Patients meeting the following criteria were included in this study: they received at least eight continuous weeks of hemodialysis exclusively through a catheter between August 1, 2004, and December 31, 2006; either catheter hemodialysis was their first treatment modality following diagnosis of ESRD (catheter as first access), or catheter hemodialysis immediately followed at least one month during which the patient was dialyzed exclusively through an arteriovenous fistula or graft (catheter as replacement access); in the first eight weeks of catheter dialysis, they did not have a gap between two consecutive outpatient dialysis sessions >30 days during which time they were not hospitalized; they had both Part A and Part B Medicare coverage during the entire catheter dialysis period; they did not have a kidney transplant during the entire catheter dialysis period; at least 95% of their catheter dialysis sessions had actual and planned blood flow rates between 100 mL/min and 500 mL/min; they were alive and in the data set for at least 90 days following the first catheter dialysis session. Planned and actual BFR values <100 mL/min or >500 mL/min were set to missing to minimize the potential impact of coding errors. In the final cohort, 99.9% of BFR values were within this range. The observation period was defined as beginning at the first catheter dialysis session and ending at the last catheter dialysis session that was uninterrupted by either a change in access or dialysis modality.

### 2.3. Variables

Using DaVita data, we reconstructed a longitudinal history of catheter dialysis treatments for each patient during their observation period. Reasons for reaching the end of the observation period were defined as (a) death, if the patient died on or before December 31, 2006, and if the last catheter dialysis session was within 30 days of death, (b) end of data (censored), if the last catheter dialysis session was within 30 days of December 31, 2006, or (c) change in access type or modality, if the last observed catheter dialysis session was not due to either death or the end of the data.

The primary outcome variable was catheter dysfunction, which was defined as actual BFR <300 mL/min despite a planned BFR ≥300 mL/min. Actual BFR was measured approximately one hour after the beginning of the dialysis session. We elected to make our definition of catheter dysfunction more restrictive than in the NKF/KDOQI clinical practice guidelines for vascular access to eliminate misclassification of catheter dysfunction where the intent, as indicated by planned BFR, was to provide BFR <300 mL/min.

Medical resource and cost outcome variables in this study were total direct medical costs to Medicare, missed dialysis sessions due to access problems, access-related procedures, and all-cause hospitalization. The DaVita data contained a record for each missed session. Each record had the date of the missed session and the reason for the missed session, including “access problems.” Access-related procedures were identified using the Medicare claims data, based on the following Health Care Common Procedure Coding System (HCPCS) codes: injection for catheter evaluation with fluoroscopy (36598); thrombolytic declotting of catheter (36593); mechanical removal of clot (36596); mechanical removal of intraluminal (intracatheter) obstructive material (75902); injection of “TPA” (J2997); tunneled catheter exchange or replacement (36581); the combination of removal of tunneled catheter (36589) *plus* tunneled catheter insertion (36558). Hospitalizations consisted of all acute care admissions for any reason and were identified from the Medicare claims.

### 2.4. Analyses

Patients were described according to their demographic and clinical characteristics at the time they began catheter dialysis, including age, gender, race, underlying cause of renal failure, dialysis vintage, ESRD network, Charlson Comorbidity Index [12], and whether catheter was their first dialysis access or a replacement for a graft or fistula. Multivariate analysis with Cox's proportional hazards model was used to examine adjusted associations between patient factors and the risk of catheter dysfunction. For the medical resource and cost analyses, patients with at least one catheter dysfunction session were divided into quintiles based on the number of catheter dysfunction sessions per month on catheter dialysis. Medical resource use and costs were compared, unadjusted, across the five groups. Analysis file construction and all analyses were performed in SAS (version 9.1.3) [13].

## 3. Results

There were 3,364 patients who met the inclusion and exclusion criteria. The average age was 62 years, 51% were male gender, 42% were black race, 46% had diabetes, and 28% had hypertension reported as the underlying cause of renal failure (Table 1). Patients with catheter as a replacement access were older, had been diagnosed with ESRD for a longer period of time, and had higher Charlson Comorbidity Index scores [12]. Those with catheter as first access were more likely to be white race. Within the catheter as replacement group, patients with catheter replacing a graft were older, had higher Charlson Comorbidity Index scores, and were more likely to be female gender and black race, compared to those with catheter replacing a fistula.

**Figure 1:**
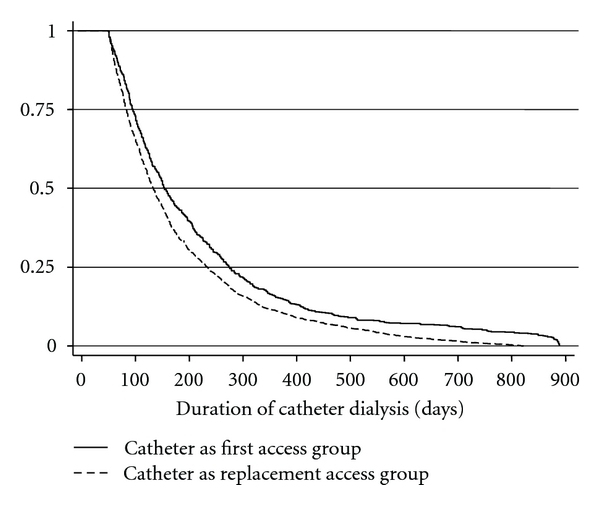
Duration of catheter dialysis.

Overall, the median duration of catheter dialysis in the cohort was 143 days, the first 56 days of which were mandated by the eligibility criteria. The median duration of catheter dialysis was significantly longer for those with catheter as first access compared to replacement access (159 days versus 138 days: *P* < 0.0001 by Log Rank) (Figure 1). Among those with catheter as a replacement access, there was no difference between those with prior graft (median 139 days) and those with prior fistula (median 136 days: *P* = 0.84 by Log Rank).

**Figure 2:**
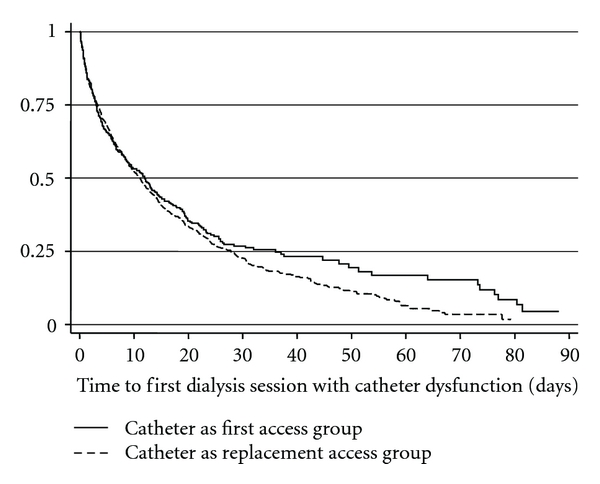
Time to first dialysis session with catheter dysfunction. Catheter dysfunction was defined as unplanned blood flow rate during the dialysis session of <300 mL/min.

**Figure 3:**
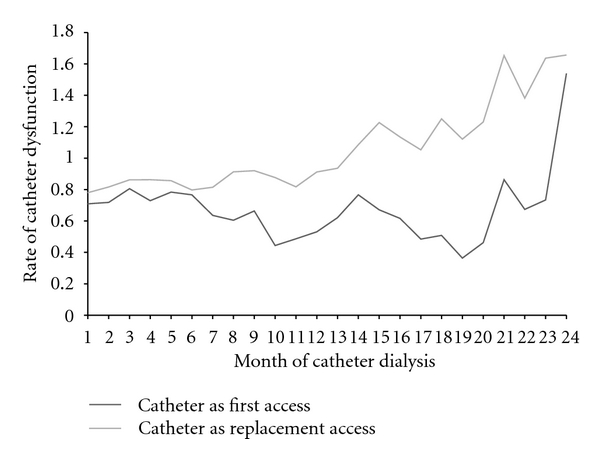
Rate of catheter dysfunction. Catheter dysfunction was defined as unplanned blood flow rate during the dialysis session of <300 mL/min.

**Figure 4:**
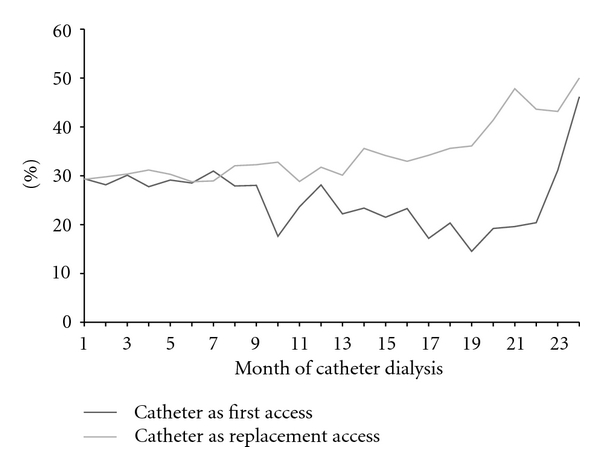
Percent of patients with catheter dysfunction. Catheter dysfunction was defined as unplanned blood flow rate during the dialysis session of <300 mL/min.

**Figure 5:**
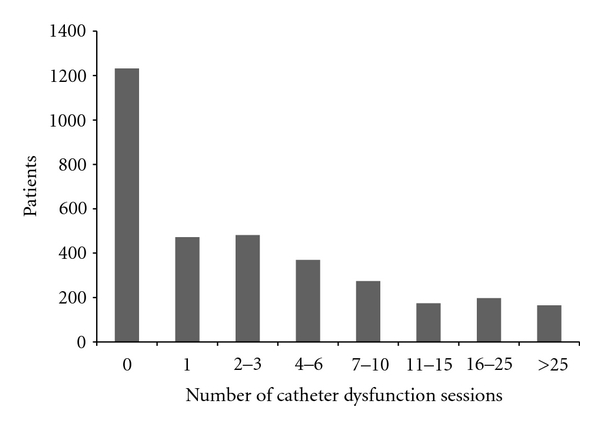
Frequency of catheter dysfunction Sessions. Catheter dysfunction was defined as unplanned blood flow rate during the dialysis session of <300 mL/min.

The cohort accounted for more than 23,000 patient-months of catheter dialysis, with the majority in the catheter as replacement access group (Table 2). Almost two-thirds of patients had at least one dialysis session with an unplanned BFR <300 mL/min, the study definition of catheter dysfunction, during their entire catheter dialysis history. Proportions were similar between catheter as first and catheter as replacement access and between prior graft and prior fistula in the catheter replacement access group. The median time to first dialysis session with unplanned BFR <300 mL/min was longer, but not significantly longer (*P* = 0.08 by Log Rank) in the catheter as first access group (Figure 2). Also the rate per patient month at risk (Figure 3) and the percent of patients with at least one session per month with unplanned BFR <300 mL/min (Figure 4) were lower in the catheter as first access group, especially after the first 9 months of catheter dialysis. Patients with at least one session meeting the study definition of catheter dysfunction had, on average, nine sessions with unplanned BFR <300 mL/min. (Table 2; Figure 5).

In the multivariate analysis using Cox's proportional hazards model for time to first session with catheter dysfunction, male gender and black race (compared to white) were associated with lower rates of catheter dysfunction (Table 3). Catheter as replacement access following graft or fistula was associated with a significantly higher rate of catheter dysfunction than catheter as first access. There was also considerable variability in the rate of catheter dysfunction across ESRD networks.

Patients in the highest quintile of catheter dysfunction sessions per month had higher average monthly direct medical costs, more missed sessions due to access problems, and more access-related procedures than those in the lowest quintile (Table 4). Hospitalization rates were similar across the five groups.

## 4. Discussion

Little has been reported on the epidemiology of hemodialysis catheter dysfunction, commonly defined, including in NKF-KDOQI guidelines, as BFR <300 mL/min. Without understanding the epidemiology of catheter dysfunction so defined, it is not possible to assess the overall impact of the NKF-KDOQI guideline on clinical and economic outcomes. Presently, data on BFR are not collected within the USRDS, or as part of CMS's ESRD Clinical Performance Measures Project. Therefore, DaVita clinical data linked to Medicare administrative, and claims data provided a unique opportunity to examine catheter dysfunction in this population. We conducted an observational study in a large cohort of patients who accounted for more than 23,000 months of catheter dialysis and 268,000 catheter dialysis sessions. Although not intended to be a random sample of all ESRD patients undergoing catheter dialysis in the United States, our cohort was geographically diverse and demographically similar to the prevalent ESRD population in 2004, the year our study began.

Our findings indicate that unplanned BFR <300 mL/min is common, occurring at least once in almost two-thirds of patients who undergo catheter dialysis for more than eight weeks, and occurring in approximately one-third of patients every month. Further, the findings indicate that unplanned BFR <300 mL/min occurs in approximately 7% of all catheter dialysis sessions. Having catheter access as a replacement for a graft or fistula is associated with a higher risk of catheter dysfunction compared to catheter as the first type of access for dialysis. Also, having more catheter dysfunction sessions per month is associated with higher direct medical costs, interruptions or delays in dialysis services, and higher rates of catheter access procedures.

There are several possible reasons why the rates we observed may underestimate the true rates. First, we excluded sessions in which planned or actual BFR values were either <100 mL/min or >500 mL/min. We did so to avoid misclassifying as catheter dysfunction sessions in which BFR values were misreported in the data. Second, although NKF/KDOQI defines catheter dysfunction as BFR <300 mL/min, our definition was more conservative because we also required the planned BFR to be ≥300 mL/min. Third, actual BFR was obtained from a single measurement approximately one hour after the beginning of the session. Fourth, by requiring patients to have at least eight weeks of catheter dialysis and to have survived at least 90 days following the start of dialysis, we excluded patients who were on catheter dialysis for shorter periods of time or who died within 90 days of beginning catheter dialysis. To the extent that catheter dysfunction is more common sooner after placement, by excluding patients with short-term catheter dialysis, we may have underestimated the overall rate of dysfunction. Catheter dysfunction due to mechanical reasons, which is known to occur sooner rather than later after placement, may be disproportionately underrepresented. Also, if death during the first 90 days after catheter placement is related to serious complications of catheter dysfunction, such as bloodstream infection, by requiring at least 90-day survival, we may have underestimated the impact of catheter dysfunction on the use of medical services, in particular on all-cause hospitalization.

In spite of these limitations, our findings suggest that catheter hemodialysis BFR <300 mL/min is common in routine dialysis care and that it impacts on the provision of dialysis services, as well as catheter-related procedures and medical costs. It is uncertain how much of the disruption in dialysis services and utilization of medical services was based solely on the perceived need to maintain BFR >300 mL/min to ensure adequate dialysis and how much was based on observed problems with dialysis adequacy.

## Figures and Tables

**Table 1:** Patient characteristics.

	Type of catheter use						
	All		First access		Replacement access		*P* value
	*N* = 3,364		*n* = 718		*n* = 2,646		
	Count	%	Count	%	Count	%	
Age							
18–49	712	21.2	150	20.9	562	21.2	<0.001
50–64	1,112	33.1	209	29.1	903	34.1	
65–74	813	24.2	165	23	648	24.5	
≥75	727	21.6	194	27	533	20.1	
Gender							
Male	1,728	51.4	366	51	1,362	51.5	0.81
Female	1,636	48.6	352	49	1,284	48.5	
Race							
White	1,694	50.4	455	63.4	1,239	46.8	<0.0001
Black	1,417	42.1	222	30.9	1,195	45.2	
Other	253	7.5	41	5.7	212	8	
Underlying cause of renal failure							
Diabetes	1,533	45.6	329	45.8	1,204	45.5	0.09
Hypertension	957	28.4	200	27.9	757	28.6	
Glomerulonephritis	341	10.1	59	8.2	282	10.7	
Other	533	15.8	130	18.1	403	15.2	
Charlson Comorbidity Index							
0	1,239	36.8	332	46.2	907	34.3	<0.0001
1-2	716	21.3	160	22.3	556	21	
3-4	779	23.2	128	17.8	651	24.6	
≥5	630	18.7	98	13.6	532	20.1	
Dialysis network							
01 New England/02 (NY)	121	3.6	24	3.3	97	3.7	<0.001
03 (NJ)/04 (PA)	128	3.8	30	4.2	98	3.7	
05 (VA)	350	10.4	75	10.4	275	10.4	
06 (NC)/08 (MS)	460	13.7	66	9.2	394	14.9	
07 (FL)	242	7.2	63	8.8	179	6.8	
09 (IN)	130	3.9	35	4.9	95	3.6	
10 (IL)	101	3	24	3.3	77	2.9	
11 (MN)	268	8	72	10	196	7.4	
12 (MO)	115	3.4	38	5.3	77	2.9	
13 (OK)	134	4	33	4.6	101	3.8	
14 (TX)	416	12.4	76	10.6	340	12.8	
15 (CO)	231	6.9	50	7	181	6.8	
16 (WA)/17 (N-CA)	311	9.2	60	8.4	251	9.5	
18 (S-CA)	357	10.6	72	10	285	10.8	

**Table 2:** Catheter dysfunction*.

	All patients *N* = 3, 364	Catheter as first access *N* = 718	Catheter as replacement access		
			All *N* = 2,646	Graft *N* = 1, 758	Fistula *N* = 888
Patient months	23,045	5,663	17,382	11,579	5,803
Catheter sessions	268,363	66,285	202,078	134,250	67,828
Patients with ≥1 CD session	2,132	454	1,678	1,123	555
% Patients with ≥1 CD session	63%	63%	63%	64%	63%
Median time (days) to 1st CD session	95	105	94	89	108
Total CD sessions	19,118	4,024	15,094	10,085	5,009
Rate of CD per patient month at risk	0.83	0.71	0.87	0.87	0.86
Mean # CD sessions among patients with ≥1 CD session	9.0	8.9	9.0	9.0	9.0

*Catheter dysfunction was defined as unplanned blood flow rate during the dialysis session of <300 mL/min.

CD: catheter dysfunction.

**Table 3:** Factors associated with catheter dysfunction*.

	Hazard ratio	95% confidence interval		*P* value
Age				
<50		Reference		
50–64	1.08	0.95	1.23	0.23
65–74	1.14	0.99	1.30	0.07
≥75	1.13	0.98	1.31	0.09
Gender				
Female		Reference		
Male	0.85	0.78	0.92	<0.0001
Race				
White		Reference		
Black	0.90	0.82	1.00	0.04
Other	0.98	0.83	1.17	0.84
Underlying cause of renal failure				
Diabetes		Reference		
Hypertension	1.08	0.97	1.20	0.17
Glomerulonephritis	0.99	0.85	1.16	0.94
Other/unknown	1.01	0.88	1.15	0.95
Network				
6 and 8		Reference		
1 and 2	0.52	0.40	0.68	<0.0001
3	0.67	0.51	0.87	<0.01
5	0.64	0.54	0.76	<0.0001
7	0.53	0.43	0.65	<0.0001
9	1.21	0.96	1.52	0.10
10	1.23	0.95	1.57	0.11
11	0.74	0.61	0.89	<0.01
12	0.70	0.54	0.91	<0.01
13	0.95	0.75	1.19	0.63
14	0.89	0.76	1.05	0.16
15	0.78	0.64	0.96	0.02
16	0.77	0.64	0.92	<0.01
18	0.44	0.36	0.54	<.0001
Charlson Comorbidity Index				
0		Reference		
1-2	0.87	0.71	1.06	0.16
3-4	0.91	0.76	1.07	0.25
>4	0.93	0.79	1.10	0.40
Replacement access				
Catheter as first access		Reference		
Catheter as replacement access	1.13	1.01	1.27	0.04

**Table 4:** Cost and resource use by number of catheter dysfunction sessions*.

Catheter dysfunction sessions/month (quintile)	Patients (*n*)	Cost and Resource Use			
		Direct medical cost**	Missed sessions due to access***	Hospitalization***	Catheter access procedure***
1 (0.04–0.32)	424	$5,390	1.2	19.2	21.3
2 (0.33–0.56)	426	$5,746	1.4	25.8	26.7
3 (0.57–1.13)	430	$5,634	1.9	23	29.9
4 (1.14–2.33)	433	$6,010	2.3	26.4	42.4
5 (>2.33)	419	$6,226	2.8	26.6	56.4
All	2,132	$5,801	1.9	24.2	35.3

*Catheter dysfunction was defined as unplanned blood flow rate during the dialysis session of <300 mL/min.

**Per month on catheter dialysis.

***Per 100 patient months at risk.
